# Lead poisoning and its clinical manifestation in opium users: a case series

**DOI:** 10.1186/s13256-026-06419-8

**Published:** 2026-10-29

**Authors:** Fatemeh Baberi, Sayed Aliakbar Banihashemi, Saeed Yousefinejad, Fatemeh Rezaei, Saba Sahraian, Alireza Mirahmadizadeh

**Affiliations:** 1 Department of Epidemiology, School of Health Sciences, Shiraz University of Medical Sciences, Shiraz, Iran; 2 Faculty of Medicine, Islamic Azad University, Kazerun, Iran; 3 Department of Occupational Health and Safety Engineering, Shiraz University of Medical Sciences, Shiraz, Iran; 4 Research Center for Social Determinants of Health, Jahrom University of Medical Sciences, Jahrom, Iran; 5 Department of Neural Science, College of Arts and Science, New York University, New York, NY USA; 6 Non-Communicable Diseases Research Center, Shiraz University of Medical Sciences, Shiraz, Iran

**Keywords:** Opium, Lead poisoning, Abdominal pain, Central nervous system, Case series

## Abstract

**Background:**

The practice of adding lead to opium to increase its weight resulted in lead poisoning among opium user, especially those who consume it orally. This study aims to examine patients with symptoms of lead poisoning who are opium users.

**Case presentation:**

We retrospectively investigated the records of 20 Iranian patients who were opium users and presented with symptoms and signs of lead poisoning. Regarding the route of opium administration, 3 patients (15%) reported smoking only, 2 patients (10%) reported oral ingestion only, and the majority of cases (15 patients, 75%) reported combined use of smoking and oral ingestion. All were married and 85% were male, with a mean age of 57.8. The average duration of opium dependence was 19.7 years, with a mean daily consumption of 2.1 g. They reported severe abdominal pain, with or without central nervous system symptoms. Blood lead levels in the patients ranged from 30 to 833 µg/dL, with two patients exceeding 600 µg/dL. Chelation therapy was used for 80% of patients, resulting in 45% having full recovery, 40% partial recovery, and a fatality rate of 15%.

**Conclusions:**

This case series emphasizes the importance of awareness among both opium users and medical staff regarding the risks related to lead poisoning due to contaminated opium. Patients should be informed about the potential contamination of opium, which can cause serious gastrointestinal and neurological symptoms, and even death. Healthcare providers should maintain a high index of suspicion for lead poisoning in opium-addicted patients presenting with compatible symptoms, ensuring early diagnosis and timely treatment, such as chelation therapy.

## Introduction

Opium consumption is the second most common drug use disorder worldwide and is especially prevalent in Middle Eastern countries [1, 2]. According to the World Drug Report 2023, the global prevalence of the use of opiates is 0.6% [3]. In South Asia, countries such as Iran, Pakistan, Afghanistan and India have historically used opium for recreational and medical purposes [3]. In Iran, substance abuse, including opium and its derivatives, not only have imposed numerous negative effects on society, including a death rate of 38.17 per million [4], but also disproportionately affects youth, making this issue a continuous concern for policymakers[5]. Iran has a long history of opium use and it remains the most commonly abused substance in the country [6]. According to the World Health Organization (WHO), Iran has the highest rate of opium abusers worldwide [7]. Opium can be consumed orally or inhaled after heating [8].

Opium addiction is recognized as one of the newest non-occupational sources of Lead poisoning in Iran [9–13]. Smugglers add lead to opium to increase weight and maximize profits ultimately, maximize profits [14, 15] a practice commonly referred to as 'stepping up' or 'blowing up,’ where weight is increased and purity is decreased [16]. Lead is a toxic metal that affects multiple body organs and their functions leading to severe clinical manifestations and even death [17]. Lead poisoning due to consumption of opium is an emerging health concern in Iran [18–20]. Oral use, particularly when individuals are unable to smoke opium—such as during travel, at work, or when attempting to conceal their addiction—may increase exposure to lead‑adulterated opium [21]. Complications such as abdominal pain, anemia and nephropathy have been reported in individuals who consume opium, likely as a result of lead contamination [22, 23]. In a study, the most common symptoms were gastrointestinal disturbances, followed by musculoskeletal complaints, including muscle weakness [16, 18, 20].

Over a 2-month period in 2017, the medical staff of a hospital in Shiraz observed a noticeable increase in cases of lead poisoning among opium users. This study aims to investigate lead poisoning prevalence, describe the demographic characteristics and clinical presentations, and assess the blood lead levels of affected individuals. As the number of cases is relatively large considering this short time period, findings of this study may offer critical insights for individuals with opium use disorders, as well as for healthcare providers and drug control authorities.

1. Case series: This study examines 20 Iranian cases of lead poisoning among patients who presented to a hospital in Shiraz. A structured questionnaire was used to collect patient information in three main categories
2. Demographics and opium use: This section included information on age, gender, educational level, occupational exposure to lead, route of opium consumption, daily intake, and duration of use.
3. Clinical presentation: clinical symptoms were categorized into two subgroups:
4. Gastrointestinal symptoms: Acute or chronic abdominal pain, Anorexia, Constipation (defined as fewer than one bowel movement per day), weight loss (defined as more than 10 kg in three months or at least 5% of total body mass over six months), and emesis
5. Central nervous system (CNS) symptoms: Decreased level of consciousness (Glasgow Coma Scale < 14), motor weakness (muscle strength < 4 on a 0–5 scale), anxiety, headache, convulsions.
6. Laboratory investigations: This included Complete Blood Count (CBC), Liver and kidney function tests, Serum electrolytes, blood glucose levels and arterial blood gas indices. A 3-mL whole blood sample was obtained through venipuncture, stored in sterile EDTA coated tubes, and analyzed for blood lead level (BLL) by graphite furnace atomic absorption spectrophotometry. BLL values were reported in micrograms per deciliter (µg/dL).

## Case definition

In this study, cases were defined as individuals with opioid dependence who reported opium use for more than 1.5 years. Patients presented with acute abdominal pain with or without central nervous system (CNS) manifestations, where clinical, physical examination findings (for example, hematologic, X-ray and CT scans) and para-clinical investigations failed to yield sufficient clues for a specific medical or surgical diagnosis. These individuals were identified as high risk and assessed for possible lead intoxication. All patients were of Iranian ethnicity.

### Inclusion criteria

Patients with a history of opium addiction, a BLL > 10 µg/dL, at least one gastrointestinal or CNS symptom of lead intoxication were defined as cases and included in this retrospective analysis in this study.

### Data analysis

Data were organized and recoded using Microsoft Excel. To describe findings, summary statistics for quantitative and categorical variables were used. These measures included minimum, maximum, arithmetic mean, standard deviation, median, and interquartile range for continuous variables and frequencies and percentages (*n*, %) for categorical variables. No hypothesis testing was conducted, since this study was designed to provide a descriptive analysis for future comparative studies.

## Results

We analyzed the data on patients’ demographic characteristics, occupational history and routes for opium abuse, which is summarized in Table 1. All were married and 85% were male, with a mean age of 57.8 ± 12.3. The majority of the patients were either laborers or drivers with less than high school education. Two subjects had possible occupational exposure to lead contaminated material through work involving paint and gasoline. Regarding the route of opium administration, 3 patients (15%) reported smoking only, 2 patients (10%) reported oral ingestion only, and the majority of cases (15 patients, 75%) reported combined use of smoking and oral ingestion. The duration of opium dependence ranged from 1.5 to 70 years with mean 19.7 ± 14.7 years. Opium consumption ranged from 0.5 to 10 g/day with mean 2.1 ± 2.3 g/day.

**Table 1:** Demographic and opium related variables among lead poisoning cases

Case No	Sex (M/F)	Age (year)	Opium consumption					Contact with lead contained materials	
			Yes/No	Duration (Year)	Amount grams/day	Rout of administration			
						Smoked	Swallowed	Yes/No	Type
	F	62	Yes	10	2.29	Yes	No	No	–
	M	65	Yes	20	3	Yes	Yes	No	–
	M	82	Yes	30	0.144	Yes	Yes	No	–
	M	51	Yes	25	2.29	Yes	Yes	No	–
	M	55	Yes	2	0.048	Yes	No	No	–
	M	45	Yes	15	0.5	Yes	Yes	No	–
	M	50	Yes	7	0.458	Yes	Yes	No	–
	M	46	Yes	15	10	Yes	Yes	No	–
	M	73	Yes	30	1	Yes	Yes	Yes	Gasoline
	F	77	Yes	70	2.29	Yes	No	No	–
	F	68	Yes	20	2.29	No	Yes	No	–
	M	47	Yes	30	2.29	Yes	Yes	No	–
	M	68	Yes	20	1	Yes	Yes	No	–
	M	38	Yes	15	4.58	Yes	Yes	No	–
	M	50	Yes	4	1.2	Yes	Yes	No	–
	M	55	Yes	20	2	Yes	Yes	No	–
	M	68	Yes	20	1.2	Yes	Yes	Yes	Dye
	M	45	Yes	1.5	0.2	No	Yes	No	–
	M	48	Yes	20	1.2	Yes	Yes	No	–
	M	64	Yes	20	4	Yes	Yes	No	–
Sum	85% M	57.8 ± 12.2 (38–82)	100%	19.7 ± 14.7 (1.5–70)	2.1 ± 2.3 (0.05–10)	90% Yes	85% Yes	10%	–

Table 2 presented the lead poisoning clinical manifestation and their duration (days) among patients. Among the GI manifestations, anorexia (85%) followed by aacute abdominal pain (80%) and constipation (70%) were the most common initial presentations. Emesis, and weight loss were less frequent symptoms accounting for 65% and 60% of the subjects, respectively. CNS manifestations were muscle weakness (90%), anxiety (65%) and decreased level of consciousness (45%). Additionally, 25% of patients complained from headache and 20% experienced convulsions during their stay in the emergency department. The cumulative symptom burden demonstrates that 75% of patients had at least 2 GI symptoms while 85% suffered from at least 2 CNS manifestations. The patients who reported myalgia as a non-specific symptom were 70%. Most of the patients (60%) had obvious pallor and 65% showed abdominal tenderness. All but three patients had the rest had normal respiratory findings.

**Table 2:** History of Lead poisoning related signs and symptoms, final outcome and their serum lead level among lead poisoning cases

Case No	Acute abdominal pain	chronic abdominal pain	Constipation	Weight loss	Weakness	Anxiety	Anorexia	Decrees level of consciousness	Headache	Nausea or Vomiting	Convulsion	Myalgia	Examination						Serum lead level (µg/dl)
													Dyspnea	Clinical anemia	Abdominal tenderness	Systolic Blood Pressure	Diastolic Blood Pressure	Died	
	Yes	No	Yes	Yes	Yes	Yes	Yes	No	Yes	Yes	Yes	Yes	Yes	Yes	Yes	170	100	Yes	833
	Yes	No	Yes	Yes	Yes	Yes	Yes	No	Yes	Yes	No	No	No	Yes	Yes	100	60	No	41
	No	No	No	No	Yes	No	No	Yes	No	No	No	No	No	No	No	140	90	No	39
	Yes	No	Yes	Yes	Yes	Yes	Yes	No	No	Yes	No	Yes	No	Yes	Yes	150	90	No	189
	Yes	No	No	No	Yes	No	Yes	No	No	No	No	Yes	No	No	Yes	110	70	No	35
	Yes	No	Yes	Yes	Yes	Yes	Yes	Yes	No	Yes	Yes	Yes	No	Yes	Yes	110	70	No	80
	No	No	Yes	Yes	Yes	No	No	No	No	No	No	Yes	No	Yes	No	140	80	No	91
	Yes	No	Yes	Yes	Yes	Yes	Yes	No	No	Yes	No	No	No	No	No	NA	NA	No	108
	Yes	No	Yes	Yes	Yes	Yes	Yes	Yes	No	Yes	No	Yes	No	No	Yes	180	110	No	120
	Yes	No	Yes	No	Yes	Yes	Yes	Yes	No	Yes	No	No	No	No	No	100	60	No	60.5
	Yes	No	Yes	Yes	No	Yes	Yes	Yes	No	Yes	No	Yes	No	Yes	No	100	60	Yes	650
	No	No	No	No	No	No	No	No	No	No	No	No	No	No	Yes	150	100	No	78.2
	Yes	No	Yes	Yes	Yes	Yes	Yes	Yes	Yes	Yes	Yes	Yes	No	No	No	180	110	No	63
	Yes	No	Yes	Yes	Yes	Yes	Yes	No	No	No	No	Yes	No	Yes	Yes	110	80	No	75
	Yes	No	Yes	Yes	Yes	No	Yes	Yes	No	No	No	Yes	No	Yes	Yes	120	70	No	175
	Yes	No	No	No	Yes	Yes	Yes	No	No	Yes	No	No	Yes	No	Yes	150	110	No	189
	No	No	No	No	Yes	Yes	Yes	Yes	No	No	Yes	Yes	Yes	Yes	No	120	70	No	30
	Yes	No	Yes	No	Yes	No	Yes	No	Yes	Yes	No	Yes	No	Yes	Yes	110	70	No	111
	Yes	No	Yes	No	Yes	No	Yes	Yes	Yes	Yes	No	Yes	Yes	Yes	Yes	160	95	Yes	157
	Yes	No	No	Yes	Yes	Yes	Yes	No	No	Yes	No	Yes	No	Yes	Yes	130	90	No	147
Sum	80% Yes	100% No	70% Yes	60% Yes	90% Yes	65% Yes	85% Yes	55% No	75% No	65% Yes	80% No	70% Yes	80% No	60% Yes	65% Yes	133.2 ± 27.1 (100–180)	83.4 ± 17.5 (60–110)	15% Yes	163.6 ± 206.1

BLL ranged from 30 to 833 µg/dL with a mean 163.58 ± 206.12. Two patients had significantly high BLL above 600 µg/dL leading to a skewness of mean and median measures. During hospitalization, 80% received chelation therapy. The results of hospitalization and treatment could be summarized as follows: 9 individuals (45%) have full recovery without symptoms, 40% of patients (8 individuals) discharged with partial recovery and 3 individuals died, which brought about fatality rate of 15%.

## Discussion

Lead toxicity most commonly presents with anemia and abdominal pain, and should, therefore, be considered in the differential diagnosis in patients with these symptoms to avoid unnecessary investigations and medications [17]. In this study, the diagnosis was confirmed through elevated blood lead levels. 85% of the patients who presented with symptoms of lead poisoning were male, a previous study conducted in Iran also showed that 96% of the patients presenting with lead poisoning were male [20]. The average age of the patients was 57.8 ± 12.3 years. In other Iranian studies, the average age of patients was reported as 48.6 ± 7.3 [24], 45.3 ± 9.4 [26] and 46.9 ± 10.1[25]. The results of the present study showed that the duration of opium dependence is ranged from 1.5 to 70 years. In other studies, opium dependence ranges from three months to 40 years [20] and 10 ± 17 [10, 25].

Since lead affects multiple organ systems of the body, it can have different types of signs and symptoms. The signs and symptoms of lead poisoning are mainly related to the digestive system, including abdominal pain, constipation, anorexia, nausea, and other symptoms related to joint pain [26, 27]. The most common gastrointestinal manifestation was anorexia. In another study, it was shown that anorexia is the most common gastrointestinal manifestation too [20, 28, 29]. Other common gastrointestinal symptoms that patients had included acute abdominal pain and constipation, as reported in previous literature [15, 30–32 ]. The most common symptom associated with CNS was muscle weakness. Another study also reported muscle weakness as the most common symptom [15, 20]. Other symptoms that were common in patients include anxiety, nausea or vomiting, anorexia, and myalgia. These symptoms have also been reported in other studies [9, 15, 20, 33].

In this study, the mean serum lead level (µg/dl) was 163.58 ± 207.12. In a study conducted in Tehran, the average blood lead level was reported as 145 ± 61 (range 61–323) µg/dL [20] and in another study, the average lead serum level in patients was a mean of 37.57 µg/dl [34]. In this case series study, various symptoms were observed in patients. Previous studies have shown that the symptoms are not necessarily correlated with blood lead levels, the emergence of clinical manifestations in lead poisoning depends on acuity of exposure, age, and individual susceptibility [35]. It is important to note that many of the toxic effects of lead are reversible if detected early and the source of exposure is eliminated. Eliminating the source of exposure should be the first step in treating lead poisoning. Patients are categorized as mild, moderate, and severe, based on the severity of their symptoms. Mild toxicity (10–44 mcg/dl) does not require chelation; moderate poisoning including a BLL of 45–69 may be treated even in the absence of symptoms with oral and parenteral agents. Severe poisoning that has a BLL of > 70 or the presence of encephalopathy needs parenteral chelation and is considered a medical emergency [16]. Chelators are used to facilitate the removal of lead from the body [36]. It has been reported that oral chelator therapy can be used in patients with blood lead levels > 40 µg/dl, patients with lead levels of 40–79 µg/dl should be referred for prompt medical evaluation, and patients with blood lead levels above 100 µg/dl, parenteral chelator therapy should be started for them in the hospital [37]. Also, patients should be strongly advised and referred to counseling if necessary to stop using opium either orally or smoked, and counseling classes should be held for them if necessary. A suitable approach for people addicted to opium can be the harm reduction approach. The results of lead poisoning identified and reported here are likely to be only the “tip of the iceberg” for several reasons. Patients who have a history of opium abuse should be completely checked for potential lead exposure and should be reinforced to stop the further consume.

## Limitations and strength

The study's primary strength lies in its case series design (*n* = 20), which provides valuable clinical insights and is one of the largest reports on lead poisoning from opium in the region. The findings serve to increase awareness among both users and healthcare providers, potentially leading to earlier diagnosis in patients with non-specific symptoms like abdominal pain. However, the study has several limitations, including its retrospective nature, which can result in incomplete data and potential selection bias. Importantly, detailed information regarding the exact quantity, purity, and pattern of opium consumption was not available, as exposure history was based primarily on patient self-report. This limitation precluded a dose–response analysis between opium use and lead toxicity. Information on concurrent use of other substances and potential co-exposures was also limited, and comprehensive toxicological screening beyond blood lead level measurement was not performed. Finally, the report does not provide information on the patients’ genetic and family histories or the specific source of lead contamination.

## Conclusion

This case series emphasizes the importance of awareness among both opium users and healthcare professionals regarding the risks related to lead poisoning due to contaminated opium. Patients should be informed that the opium they consume may be adulterated with lead serious gastrointestinal and neurological risks and even death. Healthcare providers should keep a high index of suspicion for lead poisoning in opium-dependent individuals presenting with symptoms consistent with toxicity, ensuring early diagnosis and timely treatment, such as chelation therapy.

## Data Availability

The corresponding author can provide the data upon request.
